# Determinants of e-Mental Health Use During COVID-19: Cross-sectional Canadian Study

**DOI:** 10.2196/39662

**Published:** 2022-11-16

**Authors:** Ellie Yu, Bowen Xu, Lydia Sequeira

**Affiliations:** 1 Canada Health Infoway Toronto, ON Canada; 2 Centre for Addiction and Mental Health Campbell Family Mental Health Research Institute Toronto, ON Canada

**Keywords:** digital health, mental health, e-Mental health, user profile, determinants, health service, use, utilization, COVID-19, pandemic, Canada, users, factors

## Abstract

**Background:**

Access to mental health treatment across Canada remains a challenge, with many reporting unmet care needs. National and provincial e-Mental health (eMH) programs have been developed over the past decade across Canada, with many more emerging during COVID-19 in an attempt to reduce barriers related to geography, isolation, transportation, physical disability, and availability.

**Objective:**

The aim of this study was to identify factors associated with the utilization of eMH services across Canada during the COVID-19 pandemic using Andersen and Newman’s framework of health service utilization.

**Methods:**

This study used data gathered from the 2021 Canadian Digital Health Survey, a cross-sectional, web-based survey of 12,052 Canadians aged 16 years and older with internet access. Bivariate associations between the use of eMH services and health service utilization factors (predisposing, enabling, illness level) of survey respondents were assessed using *χ*^2^ tests for categorical variables and *t* tests for the continuous variable. Logistic regression was used to predict the probability of using eMH services given the respondents’ predisposing, enabling, and illness-level factors while adjusting for respondents’ age and gender.

**Results:**

The proportion of eMH service users among survey respondents was small (883/12,052, 7.33%). Results from the logistic regression suggest that users of eMH services were likely to be those with regular family physician access (odds ratio [OR] 1.57, *P*=.02), living in nonrural communities (OR 1.08, *P*<.001), having undergraduate (OR 1.40, *P*=.001) or postgraduate (OR 1.48, *P*=.003) education, and being eHealth literate (OR 1.05, *P*<.001). Those with lower eMH usage were less likely to speak English at home (OR 0.06, *P*<.001).

**Conclusions:**

Our study provides empirical evidence on the impact of individual health utilization factors on the use of eMH among Canadians during the COVID-19 pandemic. Given the opportunities and promise of eMH services in increasing access to care, future digital interventions should both tailor themselves toward users of these services and consider awareness campaigns to reach nonusers. Future research should also focus on understanding the reasons behind the use and nonuse of eMH services.

## Introduction

### Challenges Within Mental Health Treatment in Canada

Mental health is defined as “a state of well-being in which an individual realizes his or her own abilities, can cope with the normal stresses of life, can work productively and is able to make a contribution to his or her own community” [1]. Mental health problems and illnesses refer to the “range of behaviors, thoughts and emotions that can result in some level of distress or impairment in areas such as school, work, social and family interactions and the ability to live independently” [2]. These problems can be present in many forms, including mood disorders (eg, depression, bipolar disorder), anxiety disorders, schizophrenia, personality disorders, or eating disorders [3]. The prevalence of mental illness across Canada is high, with 1 in 5 Canadians experiencing a mental health or addiction problem in any given year [4]. The COVID-19 pandemic further impacted the mental health of Canadians, with an increase seen in the prevalence of certain mental health disorders such as depression and anxiety [5], as well as psychological problems such as insomnia, as seen in multiple nations across Europe, North America, and Asia [6].

In terms of treatment, the continuum of mental health care delivery across Canada includes community-based care such as primary care clinics, social services, mental health and addictions service clinics, and residential services, along with acute care such as the emergency department, inpatient, or psychiatric services, with publicly funded treatment focusing on those deemed to be “medically necessary” [7]. Low-intensity care for mild-to-moderate mental illnesses, including private-practice psychotherapists, still requires Canadians to pay out of pocket or access services through private insurance plans [8].

As such, access to mental health treatment remains a challenge, with 2.3 million Canadians reporting that their mental health care needs were either partially or entirely unmet, with the most frequently reported barriers being awareness and navigation of services, the time required for accessing services, or not being able to pay for services [9]. Other barriers to accessing mental health treatment across Canada include long wait times, shortage of mental health professionals, lack of mental health service integration, cultural and language barriers, concerns about stigma, and inequities due to geography or demographics [10].

### e-Mental Health and the Canadian Landscape

e-Mental health (eMH), which refers to the use of internet and related technologies to deliver timely, effective mental health services [11], has emerged over the past decade across Canada as a method that increases the accessibility of services, including broadening the reach of services for individuals in rural and remote locations, while also allowing individuals in urban and semiurban locations to overcome barriers related to transportation, physical disability, or availability [12]. Shortcomings of eMH services also exist, as there is a “digital divide,” defined as the separation between those who readily have access to a computer or device (eg, smartphone) and the internet, and those who do not [13].

Within Canada, there exist national eMH programs (eg, Kids Help Phone, Wellness Together Canada) and provincially focused programs (eg, Bridge the gApp, Tel-jeunes, Togetherall, Bounce Back). These programs operate as part of mental health service delivery through hospital- or community-based providers, or as a component of a government’s mental health strategy [13]. Moreover, beyond these programs, there has been a proliferation of online self-management resources (including websites and mobile apps) developed by academic or private organizations, with many of these eMH interventions being used to support population mental health during the COVID-19 pandemic [14]. A recent systematic review also demonstrated strong support for the effectiveness of digital cognitive behavior therapy to treat insomnia [15].

COVID-19 has also accelerated the pace of adoption of digital technologies for publicly funded mental health service delivery through the implementation of virtual billing codes and an increase in access to virtual assessments [16]. Toolkits to accelerate the awareness, uptake, and implementation of services across practitioners have emerged [17,18], aiding with the awareness and adoption of eMH services.

Despite this vast variety of services available for both population-level mental health management and focused-condition or diagnosis-specific care, the utility of eMH services across Canada is not well understood. Understanding help-seeking behaviors and service utilization is critical to assessing if and how the current eMH landscape addresses current mental health needs. Andersen and Newman’s [19,20] framework states that an individual’s health service utilization is dependent on the predisposition of the individual to use services, the individual’s ability to secure services, and the individual’s illness level. Predisposing factors include demographics (eg, age, gender), social structure (eg, education, ethnicity), and beliefs, whereby some individuals have a propensity to use services more than others. Enabling factors include family factors (eg, income, insurance) and community factors (eg, urban-rural), which are conditions that make health service resources more available to an individual. Finally, illness levels include an individual’s symptoms and diagnoses, which usually represent the most immediate causes of health service use [19,20].

### Study Aims

The objective of this study was to identify predisposing, enabling, and illness-level factors associated with the utilization of eMH services across Canada during the COVID-19 pandemic, using information collected through the 2021 Canadian Digital Health Survey.

## Methods

### Recruitment and Data Collection

The study used data gathered from the 2021 Canadian Digital Health Survey, a cross-sectional, web-based survey of 12,052 Canadians aged 16 years and older with internet access. The survey was commissioned by Canada Health Infoway (Infoway) and conducted by Léger. Data collection took place from July 14 to August 6, 2021.

The survey collected information on the use of digital health services, use of health services, and socioeconomic and demographic factors of selected Canadians. Survey participants were selected from the Léger Opinion panel—the largest Canadian panel with approximately 500,000 representative panelists from all regions of Canada—using random digital dial sampling.

Respondents from hard-to-reach target groups (eg, cancer patients) were added to the panel through targeted recruitment campaigns. Administration of the survey was conducted by Léger; however, testing of the online survey was conducted by both Infoway and Léger staff. Small monetary incentives were offered to survey participants as part of the data collection process, administered through Léger. Based on respondents’ default language of choice, the survey was presented in either English or French.

### Ethics Considerations

Informed consent was collected at the beginning of the survey and no personal identifiers were collected as part of the survey. Data were coded by Léger and then transferred to Infoway for analysis. Survey data and interactive visualizations are publicly available [21]. Due to the use of publicly available data, the requirement for ethics approval was waived.

### Independent and Dependent Variables

#### Overview

Table 1 provides an overview of the independent and dependent variables used for analysis, categorized according to Andersen and Newman’s [19,20] framework for individual determinants of health service utilization.

**Table 1:** Dependent and independent variables categorized according to Andersen and Newman’s [19,20] framework for individual determinants of health service utilization.

Framework category					Variables
**Independent variables**					
	**Predisposing factors**				
		Demographics	Age, gender		
		Social structure	Ethnicity, education level, immigrant status, language, employment		
		Beliefs	eHealth literacy		
	**Enabling factors**				
		Family	Household income, health insurance coverage		
		Community	Community size, access to family doctor		
	Illness levels			Self-rated mental health status, self-rated health status, diagnosed mental health condition, caregiving status	
Dependent variable					Use of e-Mental health services

#### Independent Variables

##### Predisposing Factors

Predisposing factors indicate the propensity for an individual to use services more than other individuals based on their characteristics that exist prior to onset of any specific illness [19,20]. As per Andersen and Newman [19,20], demographics and social structures are characteristics that might predict use of health services, and are closely linked to the third component of predisposing conditions: attitudes or beliefs about care and illness.

Demographics included age and gender. Age was calculated as the difference between a respondent’s year of birth and the survey date. Gender was measured with the question, “How would you describe your gender identity?” with responses collapsed into female, male, and other.

Social structure included ethnicity, education level, immigrant status, language, and employment. Ethnicity was collected based on the question, “Which ancestry category best describes you?” and responses were dichotomized into white and nonwhite. Education level was assessed with the self-reported highest level of education obtained, including qualifications obtained outside of Canada. The categories were collapsed into high school or equivalent, college or trades, undergraduate degree, postgraduate degree, and other/none/prefer not to say. Immigrant status was based on the question, “Are you a Canadian citizen?” Language at home was based on the question “Which language do you speak on a regular basis at home?” Employment status was based on the question “What is your current employment status?” and responses were collapsed into working, unemployed, retired, disabled, student, and other/prefer not to say. Respondents who were employed either full or part time were classified as employed.

eHealth literacy was measured using the eHealth Literacy Scale (eHEALS), an 8-item self-assessment tool designed to measure respondents’ knowledge, comfort, and perceived skills at finding, evaluating, and applying electronic health information to health problems [13]. Originally developed to assess eHealth literacy levels among youth and youth workers by Skinner and Norman [13], the scale has since been adapted to a variety of settings, population groups, and multiple languages [14]. Each question measures an aspect of perceived eHealth literacy and is scored on a Likert scale ranging from 1 to 5. Scores are summed to derive an overall eHealth literacy score that ranges from 8 to 40 for each respondent. A higher eHEALS score represents higher self-perceived eHealth literacy.

##### Enabling Factors

Despite individuals being predisposed to using health services, it is necessary to have the means available for them to access these services. Enabling conditions are those that make health service resources available to individuals [19,20], which were broken down into family and community variables in this study.

With regard to family, household income was based on the total self-reported household income before tax in the past year. The categories were collapsed into $24,999 or less, $25,000-$80,000, $80,000 or more, and prefer not to answer (in CAD, in which CAD $1=US $0.78 at the time of the survey). Insurance coverage was based on the question “Which of the following best describes the type of health insurance coverage you currently have?” and the categories were collapsed into public coverage only, private coverage, no coverage, and don’t know/prefer not to say. Private coverage includes insurance plans paid for by the respondent, a family member, an employer, or an association.

With regard to community, access to a family doctor was assessed through the question “Do you have a family doctor or regular place of care, such as a health center or a family medical/medicine group?” The responses were dichotomized into yes and no/don’t know.

Community size was based on the question “How would you describe the community you live in?” and responses were collapsed into rural, small to large population centers, and urban center. The population size for a rural community was defined as less than 1000 people. The population size for a small, medium, and large population center was defined as 1000-29,999 people, 20,000-99,999 people, and 100,000-999,999 people, respectively. Urban centers were defined as 1 million people and over.

##### Illness Levels

Illness-level factors represent the most immediate cause of utilization of health services and can include perspectives of illness as well as clinical diagnoses [19,20]. Self-reported mental health (SRMH) status and self-reported health (SRH) status were measured by asking respondents, “In general, how would you rate your overall physical/mental health?” The responses were collapsed into fair/poor, good, very good/excellent, and prefer not to say. Self-reported diagnosed mental health condition was assessed with the question “Do you have emotional, psychological, or mental health conditions (eg, anxiety, depression, bipolar disorder, substance abuse, anorexia, etc) diagnosed by a health professional?” The responses were dichotomized into yes and no.

Caregiver status was based on the question, “Do you have primary or joint responsibility for providing care and/or assistance to someone?” Respondents were given the prompt that assistance refers to voluntary assistance, excluding employment or work done for payment.

#### Outcome (Dependent) Variable

The dependent variable was the use of eMH services, which was measured with the question, “Did you access websites, mobile applications (apps) or interactive online tools and services to help or support you with mental health issues you may be dealing with, such as depression, anxiety, or substance abuse in the last 12 months?” Responses were dichotomized into yes and no/don’t know.

### Statistical Analysis

#### Participant Profile

SPSS version 24 (IBM SPSS Statistics) was used for descriptive analyses. SAS version 9.4 (SAS Institute Inc) was used for logistic regression analyses. All estimates reported are based on weighted data that reflect the age, gender, and geographic distribution of Canadians aged 16 years and above in the 2016 census. Descriptive statistics were calculated for respondents who had used eMH services during the past 12 months and those who had not. Cross-tabulations were used to estimate the prevalence of eMH service utilization within our sample as well as characteristics associated with users and nonusers of eMH services.

#### Bivariate Associations for Use and Nonuse of eMH Services

Bivariate associations between the use of eMH services and predisposing, enabling, and illness-level factors of survey respondents were assessed using *χ*^2^ tests for categorical variables and *t* tests for the continuous variable.

#### Unadjusted Logistic Regression Model to Assess Determinants of Use of eMH Services

Two independent adjusted logistic regression models were performed. The first model was adjusted for predisposing, enabling, and illness-level factors. Predisposing factors included in the model were ethnicity, education level, immigrant status, home language, employment status, and eHealth literacy. Enabling factors included in the model were household income, health insurance coverage, access to family doctors, and community size. Illness-level factors included in the model were SRMH, SRH, diagnosed mental health status, and caregiver status.

#### Adjusted Logistic Regression Model to Assess Determinants of Use of eMH Services

The second model was also adjusted for predisposing, enabling, and illness-level factors along with demographic factors, including age and gender. The adjusted multivariable logistic regressions were performed to assess associations between predisposing, enabling, and illness-level factors and use of eMH services controlling for age and gender.

We tested for multicollinearity by assessing the bivariate correlation between two predictor variables. No interactions were found between access to a family physician, SRH, SRMH, diagnosed mental health condition, household income, education, immigrant status, language at home, employment status, insurance coverage, age, gender, ethnicity, community size, and caregiver status.

## Results

### Participant Profile

A total of 12,052 Canadians aged 16 years or older were surveyed. The proportion of respondents who self-reported using an eMH service in the past 12 months (ie, users) was 883 out of 12,052 (7.33%) and the proportion of respondents who did not use any eMH service in the past 12 months (ie, nonuser) was 11,169 out of 12,052 (92.67%). Table 2 compares the predisposing, enabling, and illness-level factors of users and nonusers of eMH services.

The average age of eMH users was 40.4 (SD 15.97) years and the average age of nonusers was 47.61 (SD 17.72) years. The proportion of women within our sample was higher among users of eMH services as compared to nonusers of eMH services. The proportion of those who identified in the gender category “other” varied significantly between users and nonusers, with a higher proportion falling within the eMH-user group (Table 2). In our sample, the prevalence of white respondents among users of eMH services was lower than that of nonusers. A higher percentage of users of eMH services identified as immigrants and noncitizens, as employed full or part time, disabled, and a student when compared to nonusers. The difference in the distribution of education level among users and nonusers was statistically significant, with a higher percentage of eMH users having obtained at least an undergraduate degree compared to nonusers. The percentage of eMH users living in rural communities was lower than that of nonusers, with a higher percentage of eMH users living in urban centers. A higher percentage of eMH users self-reported to be caregivers compared to nonusers. A higher percentage of eMH service users reported their annual household income to be CAD $80,000 or more compared to nonusers. A higher percentage of users of eMH services identified as having private insurance coverage when compared to nonusers.

**Table 2:** Characteristics of users and nonusers of e-Mental health (eMH) services, and associations with demographic, health-related, and socioeconomic factors for Canadians aged 16 years or older (2021 Canadian Digital Health Survey; N=12,052).

Predictor variables					Used eMH services in the past 12 months (n=883 unweighted, n=897 weighted)		Did not use eMH services in the past 12 months (n=11,169 unweighted, n=11,155 weighted)		*χ*^2^ or *F (df)*		*P* value	
**Predisposing factors**												
	Age, mean (SD)				40.40 (15.97)		47.61 (17.72)		35.06 (1)		<.001	
	**Gender, n (%)^a^**									8.78 (2)		.01
			Man (ref)		412 (45.9)		5384 (48.3)					
			Woman		467 (52.0)		5658 (50.7)					
			Other^b^		18 (2.0)		113 (1.0)					
	**Ethnicity, n (%)**									24.24 (1)		<.001
		White		608 (67.8)		8390 (75.2)						
		Nonwhite^b,^^c^ (ref)		289 (32.30)		2765 (24.8)						
	**Education level, n (%)**									43.22 (3)		<.001
		High school or equivalent (ref)		181 (20.2)		2537 (22.7)						
			College or trades		197 (21.9)		3231 (29.0)					
			Undergraduate degree^b^		365 (40.7)		3668 (32.9)					
		Postgraduate degree^b^		136 (15.2)		1342 (12.0)						
		Other/none of above/prefer not to answer		18 (2.5)		377 (3.4)						
	**Immigrant status, n (%)**									2.21 (2)		.33
		Born in Canada (ref)		713 (79.5)		9016 (80.8)						
		Immigrant^d^		137 (15.2)		1667 (14.9)						
			Not a citizen		47 (5.3)		472 (4.2)					
	**Language at home, n (%)**									55.21 (2)		<.001
			English (ref)		765 (85.3)		8267 (74.1)					
			French^b^		107 (11.9)		2,373 (21.3)					
			Other^b^		25 (2.8)		515 (4.6)					
	**Employment status, n (%)**									91.06 (5)		<.001
		Employed (full or part time) (ref)		593 (66.1)		6255 (56.1)						
		Unemployed		74 (8.3)		888 (8.0)						
		Retired^b^		92 (10.3)		2644 (23.7)						
		Disabled		30 (3.4)		291 (2.6)						
		Student		92 (10.2)		839 (7.5)						
		Other/prefer not to say		16 (1.7)		238 (2.1)						
**Enabling factors, n (%)**												
	**Household income^e^**									5.73 (3)		.13
		24,999 or less (ref)		79 (8.8)		1051 (9.4)						
		25,000-80,000		322 (35.9)		4253 (38.1)						
		80,000 or more		413 (46.0)		4690 (42.1)						
		Prefer not to answer		83 (9.3)		1161 (10.4)						
	**Insurance coverage**									10.67 (3)		.01
		Public only (ref)		285 (31.8)		3844 (34.5)						
		Private		488 (54.4)		5,465 (49.0)						
		No coverage		64 (7.1)		997 (8.9)						
		I don’t know/prefer not to say		60 (6.7)		849 (7.6)						
	**Community size**									6.03 (2)		.05
		Rural (ref)		58 (6.4)		909 (8.2)						
		Small-large population centers		585 (65.2)		7420 (66.5)						
		Urban centers		254 (28.3)		2826 (25.3)						
	**Access to a family doctor**									17.60 (1)		<.001
		Yes (ref)		825 (91.9)		9722 (87.2)						
		No/don’t know^b^		72 (8.1)		1433 (12.8)						
**Illness levels, n (%)**												
	**SRMH^f^**									154.21 (3)		<.001
		Excellent/very good (ref)		293 (32.7)		5164 (46.3)						
		Good		237 (26.4)		3442 (30.9)						
		Poor/fair^b^		364 (40.6)		2504 (22.4)						
		Prefer not to say		3 (0.3)		45 (0.4)						
	**SRH^g^**									6.73 (2)		.08
		Excellent/very good (ref)		374 (41.7)		4811 (43.1)						
		Good		324 (36.1)		4204 (37.7)						
		Poor/fair		198 (22.1)		2103 (18.9)						
		Prefer not to say		1 (0.1)		37 (0.3)						
	**Diagnosed mental health condition**									241.19 (1)		<.001
		Yes (ref)		312 (34.7)		1655 (14.8)						
		No^b^		585 (65.3)		9500 (85.2)						
	**Caregiver status**									114.04 (1)		<.001
		Yes (ref)		331 (36.9)		2388 (21.4)						
		No^b^		566 (63.1)		8766 (78.6)						

^a^Percentages are weighted and have been rounded, thus may not total 100.

^b^Significantly different from estimate for reference category (*P*<.05), Bonferroni-adjusted pairwise *Z*-test.

^c^Including respondents who selected “prefer not to answer” and “other.”

^d^Referring to the proportion of respondents who are immigrant and granted citizenship of Canada under the *Citizenship Act.*

^e^In Canadian dollars (CAD $1=US $0.78).

^f^SRMH: self-rated mental health status.

^g^SRH: self-rated health status.

### Bivariate Associations for Use and Nonuse of eMH Services

Table 2 outlines the bivariate associations between use of eMH service and predisposing, enabling, and illness-level factors. The average age of eMH service users was significantly younger than that of nonusers of eMH services. The association between gender and use of eMH services was also statistically significant. Education, employment, language, and insurance coverage were all significantly associated with the use of eMH services. Ethnicity was also significantly associated with the use of eMH services. The prevalence of nonwhite individuals significantly differed from that of white individuals for both users and nonusers, although the proportional difference between the prevalence of white and nonwhite respondents was more pronounced among nonusers.

Similarly, the prevalence of caregivers significantly differed from that of noncaregivers for both users and nonusers, and the proportional difference was more pronounced among nonusers. Access to a family physician was significantly associated with the use of eMH services, with a higher proportion of caregivers among those reporting using eMH services when compared to nonusers. SRMH status was also significantly associated with the use of eMH services, with significant differences between respondents who reported a fair/poor mental health status and those who reported an excellent/very good mental health status. The prevalence of a diagnosed mental health condition was significantly associated with use of eMH services, with a greater proportion of users having a self-reported mental health condition.

For education, those with an undergraduate or postgraduate degree significantly differed from those with only high school or equivalent diplomas, for both users and nonusers. For language, the prevalence of English speakers was significantly different from that of French or other-language speakers, for both users and nonusers. The only group that differed significantly from the reference group of full-time or part-time employees was retired individuals for both users and nonusers. Post hoc tests did not reveal pairwise differences for insurance coverage. 

### Unadjusted Logistic Regression Model to Assess Determinants of Use of eMH Services

Table 3 shows the estimates and odds ratios from the unadjusted regression model of predisposing, enabling, and illness-level factors and the associations with use of eMH services.

For predisposing factors, those with an undergraduate or postgraduate degree had higher odds of using eMH services when compared to those with a high school diploma or equivalent. Survey respondents who did not speak English at home had lower odds of using eMH services. Compared to respondents who were employed, those who were unemployed, retired, or disabled had lower odds of using eMH services. Finally, those with a higher eHEALS score had higher odds of using eMH services.

The only enabling factor significantly associated with eMH use was family physician access. Having a regular family doctor was positively associated with use of eMH services.

Except for SRH, all illness-level factors were significantly associated with the use of eMH services. For SRMH status, those with fair or poor SRMH were more likely to use eMH services. Having a diagnosed mental health condition and being a caregiver were both positively associated with greater odds of using eMH services.

**Table 3:** Logistic regression adjusted for predisposing, enabling, and illness-level factors and their association with e-Mental health service use among Canadians aged 16 years or older (2021 Canadian Digital Health Survey).

Variables		Odds ratio	95% CI		*P* value	
**Predisposing factors (vs reference group)**						
	Ethnicity (nonwhite vs white)	0.80	0.67-0.95		.01	
	Education (college, trades vs high school)	0.91	0.73-1.14		.43	
	Education (undergraduate degree vs high school)	1.34	1.10-1.65		.005	
	Education (postgraduate degree vs high school)	1.35	1.05-1.75		.02	
	Education (prefer not to say, none of the above, other vs high school)	0.86	0.52-1.43		.56	
	Immigrant status (immigrant, not a citizen vs born in Canada)	1.02	0.84-1.24		.85	
	Language (other than English vs English)	0.58	0.48-0.70		<.001	
	Employment (unemployed^a^ vs employed)	0.66	0.55-0.79		<.001	
	Employment (student vs employed)	1.19	0.92-1.54		.18	
	Employment (prefer not to say, other vs employed)	0.66	0.38-1.14		.14	
	eHEALS^b^	1.05	1.04-1.06		<.001	
**Enabling factors (vs reference group)**						
	Income^c^ (25,000-79,000 vs <25,000)	0.92	0.70-1.21		.56	
	Income (80,000 or more vs <25,000)	0.94	0.71-1.25		.67	
	Income (prefer not to say vs <25,000)	0.99	0.71-1.39		.96	
	Insurance (public only vs has private insurance)	0.98	0.83-1.15		.79	
	Insurance (no coverage, don’t know, prefer not to say vs has private insurance)	0.82	0.66-1.02		.08	
	Has a family physician (yes vs no, don’t know)	1.47	1.14-1.90		<.001	
	Community size (small to large population centers vs rural)	1.09	0.81-1.45		.58	
	Community size (urban center vs rural)	1.04	0.76-1.42		.82	
**Illness-level factors (vs reference group)**						
	SRMH^d^ (fair, poor vs excellent, very good, good, prefer not to say)	1.79	1.51-2.13		<.001	
	SRH^e^ (fair, poor vs excellent, very good, good, prefer not to say)	0.90	0.74-1.08		.26	
	Diagnosed mental health condition (yes vs no)	2.35	1.98-2.79		<.001	
	Caregiver status (yes vs no)	1.87	1.61-2.17		<.001	

^a^Unemployed includes disabled and retired respondents.

^b^eHEALS: eHealth Literacy Scale.

^c^Income given in Canadian dollars (CAD $1=US $0.78).

^d^SRMH: self-rated mental health.

^e^SRH: self-rated health.

### Adjusted Logistic Regression Model to Assess Determinants of Use of eMH Services

Table 4 shows the estimates of the adjusted logistic regression model of predisposing, enabling, and illness-level factors, and the associations with use of eMH services.

Education, language, and eHealth literacy were predisposing factors that significantly predicted use of eMH services. Both those with undergraduate and postgraduate education had higher odds of using eMH services compared to those with high school education. Respondents who did not speak English at home had lower odds of using eMH services compared to those who did. Again, those with a higher eHEALS score had higher odds of using eMH services.

Access to a family physician, community size, and income were enabling factors significantly associated with use of eMH services. Those making CAD $25,000-$79,000 were less likely to use eMH services than those making less than CAD $25,000; however, for those making CAD $80,000 or more or for those who did not report their income, this relationship was not significant. Those with a regular family physician and those living in small, medium, or large population centers were more likely to use eMH services than those without a family physician and those living in rural areas, respectively.

For illness-level factors, in the model adjusted for age and gender, SRMH was not significantly associated with use of eMH. Those who rated their SRH as poor or fair had lower odds of using eMH services (Table 3).

**Table 4:** Logistic regression adjusted for predisposing, enabling, illness-level, and demographic factors and their association with e-Mental health service use among Canadians aged 16 years or older (2021 Canadian Digital Health Survey).

Variables			Odds ratio		95% CI		*P* value
**Predisposing factors (vs reference group)**							
	Ethnicity (nonwhite vs white)	0.89		0.75-1.07		.22	
	Education (college, trades vs high school)	1.00		0.80-1.25		.99	
	Education (undergraduate degree vs high school)	1.40		1.14-1.72		.001	
	Education (postgraduate degree vs high school)	1.48		1.14-1.92		.003	
	Education (prefer not to say, none of the above, other vs high school)	0.84		0.50-1.40		.50	
	Immigrant status (immigrant, not a citizen vs born in Canada)	1.07		0.88-1.30		.49	
	Language (other than English vs English)	0.56		0.46-0.69		<.001	
	Employment (unemployed^a^ vs employed)	0.87		0.71-1.07		.20	
	Employment (student vs employed)	0.95		0.73-1.24		.70	
	Employment (prefer not to say, other vs employed)	0.74		0.43-1.27		.27	
	eHEALS^b^	1.05		1.03-1.06		<.001	
**Enabling factors (vs reference group)**							
	Income^c^ (25,000-79,000 vs <25,000)	0.97		0.73-1.27		<.001	
	Income (80,000 or more vs <25,000)	1.00		0.75-1.33		.80	
	Income (prefer not to say vs <25,000)	1.06		0.76-1.49		>.99	
	Insurance (public only vs has private insurance)	1.02		0.86-1.20		.73	
	Insurance (no coverage, don’t know, prefer not to say vs has private insurance)	0.77		0.62-0.96		.85	
	Has a family physician (yes vs no, don’t know)	1.57		1.22-2.03		.02	
	Community size (small to large population centers vs rural)	1.08		0.81-1.44		<.001	
	Community size (urban center vs rural)	1.05		0.77-1.43		.62	
**Illness-level factors (vs reference group)**							
	SRMH^d^ (fair, poor vs excellent, very good, good, prefer not to say)	1.68		1.41-2.00		.77	
	SRH^e^ (fair, poor vs excellent, very good, good, prefer not to say)	0.95		0.78-1.15		<.001	
	Diagnosed mental health condition (yes vs no)	2.20		1.85-2.62		.58	
	Caregiver status (yes vs no)	1.85		1.59-2.15		<.001	
**Demographic factors**							
	Gender	1.19		0.70-2.02		.52	
	Age	0.98		0.98-0.99		<.001	

^a^Unemployed includes disabled and retired respondents.

^b^eHEALS: eHealth Literacy Scale.

^c^Income given in Canadian dollars (CAD $1=US $0.78).

^d^SRHM: self-rated mental health.

^e^SRH: self-rated health.

## Discussion

### Principal Results and Comparison With Prior Work

Our results demonstrate that the adoption of eMH across Canada is limited, with only 883 out of 12,052 (7.33%) survey respondents reporting use of these services within the last 12 months. Lifetime usage of eMH was only slightly higher within this sample, at 1217 out of 12,052 (10.10%). This low usage of eMH services has been observed in previous research from Germany [22], which contrasts with data from the United States in which up to 55% of respondents reported using specific digital mental health tools and technologies to manage mental health during the COVID-19 pandemic. The difference between eMH use across Canada and the United States could in part be due to the interpretation of the term “e-Mental health,” as some individuals exclude the use of telephones within this definition and others include it. Additionally, there have been several digital health policies implemented within the United States (eg, HITECH Act, 21st Century Cures Act) that have improved the adoption of digital health across the country [23], with digital health (including mental health) across Canada inching behind. Funding models for health care also differ between the two countries [24], with many more opportunities for growth of digital health companies in the United States with private-insurance payers when compared to public-insurance payers across Canadian provinces.

Benefits of our study sample include its representativeness of the Canadian population. Moreover, within our study, 35% of individuals who reported being in very good mental health sought eMH services within the prior 12 months, demonstrating that the survey respondents were also using these tools for maintaining mental health rather than solely for treating mental illness. Previous research has identified older adults’ motivation to use technology to improve mood through mechanisms of distraction, normalization, and facilitated expression of mental states [25].

### Profile of eMH Service Users

The predisposing demographic factors that had a significant association with use of eMH services included age, where younger participants were more likely to be users of eMH services. This result is consistent with population-level trends on the use of digital health technology seen in the United States, where younger people were found to be more likely to use these forms of health care [26]. A systematic review of mental health help-seeking behaviors among young people found that facilitators for online options included greater anonymity and confidentiality (which could lower concerns related to stigma), timely access through the ability to access care 24 hours per day, and empowerment through improved information access [27].

With regard to variables related to an individual’s social structure, ethnicity and immigrant status were associated with eMH use, with the prevalence of white respondents among users of eMH services lower than that of nonusers. This higher proportion of individuals within the eMH user group could speak to the increase in access to providers who share cultural and linguistic backgrounds with immigrant participants or with participants of a particular ethnicity [28].

Finally, individuals reporting poor SRMH were 1.7 times more likely to use eMH services, and those with a diagnosis of a mental health condition were 2.5 times more likely to use these services, demonstrating Andersen and Newman’s [19,20] individual determinant of illness level, which represents the most immediate cause of health service use. However, some of these significant associations were not translated to our logistic regression analyses, a finding that requires further investigation.

### Determinants of Use of eMH Services

Through our logistic regression analyses, three of the predisposing factors that predicted eMH use included education, language, and eHealth literacy. Similar trends have been identified within research studying help-seeking behaviors of ethnic minorities with a mental health diagnosis [29], due to both attitudinal barriers and structural barriers such as the cultural inappropriateness of interventions [29,30]. Another predisposing factor, eHealth literacy, was also a significant predictor of the use of eMH services. eHealth literacy has been defined as a metaliteracy comprised of traditional literacy and numeracy, health literacy, computer literacy, science literacy, media literacy, and information literacy [31]. Similar trends have borne out in previous research on the use of Web 2.0 websites such as Facebook and Twitter for searching for and sharing health information [32]. Research has also identified the impact of eHealth literacy on differing levels of trust with digital channels and sources, with higher eHealth literacy in certain populations leading to a high perceived trust in online government organizations [33].

With regard to enabling features, those with access to a family physician were more likely to use eMH services, which could potentially be due to an increase in awareness of these services. Past research has shown that the actions of health care professionals can influence patient activation and engagement, with engagement predicting digital information–seeking behavior [34]. Primary care is a majority of Canadians’ first point of contact with the health system for mental health and addiction challenges, and also where the majority of mental health care is delivered [35,36]. With efforts to educate health professionals (including primary care) about the use and implementation of eMH resources [18], such education and awareness are likely to be passed onto patients.

Factors such as having a higher income and living within nonrural areas have proven to be enablers to accessing eMH services, furthering the evidence of the existence of a digital divide. Challenges with improving rural and remote communities’ access to broadband internet persist, and until regulatory changes happen across Canada, this gap will continue to hinder access among rural communities [37]. Beyond broadband access, the need for devices (eg, computers, tablets, smartphones) is necessary for engagement in eMH services, along with appropriate digital literacy [38].

Finally, caregiver status was a predictor of eMH use within our sample. The COVID-19 pandemic increased caregiving intensity and caregiving burden [39], which had downstream impacts on caregivers’ mental health [40].

It is evident that while there has been a proliferation of eMH services by governmental offerings and beyond, expanded by the COVID-19 pandemic, the range of eMH services is vast and service utilization remains low. In addition, empirical evidence on the effectiveness of eMH service remains limited. With rapid expansions of eMH service options and technologies, it is imperative that there will be a coordinated national approach to direct eMH policies, research, and best-practice guidelines moving forward. Nonetheless, results from our survey were able to identify a persona of eMH service users during the COVID-19 pandemic, allowing for key population-level insights. Expanding eMH care across Canada will require raising awareness about available technologies and integrating “proven” technologies within the model of care [41].

Our findings suggest that SRMH was significantly associated with use of eMH, but when controlled for age and gender there was no significance. This suggests that age and gender have a stronger effect on the use of eMH services when compared to the effect of SRMH alone. Previous literature has shown that individuals who identified as male showed significantly lower recognition of symptoms associated with mental illness [42]. Research has also demonstrated that younger individuals tended to have more positive mental health perceptions, where they are able to identify and acknowledge their mental health better than older individuals [43].

### Limitations

A limitation of our study is the imbalance in responses among participants for our dependent variable (ie, use of eMH services), with eMH users making up a very small percentage (7.33%) of the sample. Moreover, at a constant threshold of *P*<.05, large sample sizes are more likely to find a significant relationship if one exists [44]. This is why we reported the odds ratios for both unadjusted and adjusted models. Additionally, the measure that identified use of eMH services asked about individuals’ access of “websites, mobile applications, or interactive online tools,” without specifying telephone as a modality (follow-up questions listed telephone services as options). This could have caused a potential decrease in the number of individuals who identified as using these services within the last 12 months.

In addition, there were missing data for approximately 10% of the questions on income levels, where individuals had chosen the “prefer not to answer” option. This response rate is comparable to the literature, whereby questions on income are often unanswered by a small percentage of respondents [45].

We also recognize that the Canadian Digital Health Survey is an online survey and therefore may limit participation of populations with limited access to technological equipment and internet, as well as certain ethnic/culture groups overrepresented in these populations. In addition, the survey did not collect information on the duration or completion of the eMH encounter, and therefore the visit could have varied in duration, quality, and completeness. As the survey relies on self-reported data, data collected may be impacted by recall error, although past research has shown that bias and variance of recall error of health care usage were minimized for the 12-month recall period [30].

### Conclusion

Our study provides an overview of the individual determinants of eMH use across Canada. The proportion of eMH service users was small, and users were likely to be those with regular family physician access, living in nonrural communities, more educated, eHealth-literate, and English-speaking. Given the opportunities and promise of eMH services in increasing access to care, future digital interventions should both tailor themselves toward users of these services, while also considering awareness campaigns to reach nonusers. Understanding the reasons behind use and nonuse is also important.

## Abbreviations

eHEALS: eHealth Literacy Scale
eMH: e-Mental health
SRH: self-reported health
SRMH: self-reported mental health
